# To the Patrons of the Journal

**Published:** 1873

**Authors:** 


### EDITORIAL AND MISCELLANEOUS.

### TO THE PATRONS OF THE ATLANTA MEDICAL AND SURGICAL JOURNAL.

With this issue of the JOURNAL, we cease to be its publishers. The terrible stringency of the money market, and the frequently recurring disasters in the commercial world, have made it necessary that we devote so much of our time to our newspaper business as to render it impossible to give that attention to the JOURNAL which would insure its regularity of issue, and proper appearance. Hence we are forced to return it to its former publishers. We have made many friends during our connection with the JOURNAL, and desire, in retiring, to bear testimony to the pleasant relations invariably existing between ourselves and its editors. Wishing it a long course of prosperity, we are,

Very Respectfully,

ALSTON & GRADY,
*Proprietors Daily Herald.*

---

From the above notice, it will be seen that the HERALD PUBLISHING COMPANY have, after publishing the JOURNAL one year, returned the proprietorship to the undersigneed, who will, until further notice, have entire control of its business management, and will be alone authorized to receipt for dues, either for subscriptions or advertisements.

We will not attempt here to apologize for the irregularity with which the JOURNAL has been issued for the past few months, nor for the issue of the September and October numbers under the same cover, as we have had nothing to do with its business management for the past year. For the future, we are responsible, and pledge ourselves that the JOURNAL shall be regularly issued every month.

The November number is now under way, and will be sent to subscribers the last of November.

W. F. WESTMORELAND.

